# Neuropathology and neuroinflammation in Alzheimer’s disease via bidirectional lung–brain axis

**DOI:** 10.3389/fnagi.2024.1449575

**Published:** 2024-08-30

**Authors:** Jie Lu, Cheng-Jun Li, Jing Wang, Yang Wang

**Affiliations:** ^1^Department of Respiratory and Critical Care Medicine, Shenyang First People’s Hospital, Shenyang Brain Hospital, Shenyang, China; ^2^Department of Pleurisy, Shenyang Tenth People’s Hospital, Shenyang Chest Hospital, Shenyang, China

**Keywords:** Alzheimer’s disease, lung–brain axis, neuropathology, neuroinflammation, peripheral mechanism

## Abstract

Alzheimer’s disease (AD) is the most common form of age-related dementia worldwide. Although the neuropathology of AD is clear, its pathogenesis remains unclear. Recently, conceptualising AD as brain-centred has reoriented many scientists because the close functional relationship between the peripheral and central nerves is increasingly recognised. Recently, various studies have focused on the crosstalk between peripherals and centrals. A new hotspot of research and new therapeutic strategies have emerged from this great progress. This mini-review is an overview of the potential molecular mechanism in AD via the bidirectional lung-brain axis, providing a new perspective for the systemic understanding of AD onset.

## 1 Introduction

Alzheimer’s disease (AD) is one of the most common forms of age-related dementia, accounting for 60% of all confirmed cases of dementia (Leng and Edison, 2021; Knopman et al., 2021). The incidence of AD is expected to continue to increase owing to population ageing (Leng and Edison, 2021; Knopman et al., 2021). AD has become a major public health problem, causing great distress to patients and their families and a heavy economic burden on society. The main challenge for AD is the confusion over its pathogenesis, resulting in difficulties finding effective treatments. Therefore, a better understanding of the molecular mechanisms of AD could improve diagnostic and therapeutic strategies (Leng and Edison, 2021; Knopman et al., 2021).

The basic pathological features of AD include amyloid plaques, neurofibrillary tangles, neuronal degeneration in the brain, and neuroinflammation due to the activation of microglia and astrocytes (Berk et al., 2014; McKhann et al., 1984). The causes of AD are described in various hypotheses, such as the amyloid (Selkoe and Hardy, 2016), tau propagation (Lewis and Dickson, 2016), mitochondrial cascade (Zong et al., 2024), calcium homeostasis (Popugaeva et al., 2015), neurovascular (Wang et al., 2023), and inflammatory hypotheses (Leonard, 2001). However, the aetiology of AD remains unclear. Recently, the conceptualisation of neurodegenerative diseases as neuron-centred diseases has shifted the grounds for many scientists (Zhang et al., 2022). There has been increasing recognition of the close functional relationship between the peripheral system and the central nervous system (CNS) (Li et al., 2024; Sun et al., 2024), as the CNS widely communicates with organs in the peripherals, such as the intestines, liver, kidneys, and other organs (Ng et al., 2024; Manocha et al., 2019; Perényi et al., 2020).

The lungs are one of the most vital organs in the human body and are responsible for the exchange of gases and the delivery of oxygen to tissues throughout the body via the circulatory system. Although the brain is the most oxygen-consuming organ, and the physiology of the lungs and brain is well understood, the lung-brain axis is poorly understood in the pathogenesis of AD. This mini-review focuses on the neuropathology of AD via the bidirectional lung-brain axis, providing a new perspective for the systemic understanding of AD onset.

## 2 Alzheimer’s disease and lung disease share common risk factors

Cigarette smoking is a major lifestyle factor that causes lung diseases such as chronic bronchitis, emphysema, bronchiectasis, tuberculosis, and cancer (Siddiqi et al., 2023). Recently, cigarette smoking has been associated with cognitive decline and is recognised as a risk factor for AD (Durazzo et al., 2014; Kivipelto et al., 2008). Typical neuropathological changes show that long-term cigarette smokers experience a reduction in brain volume, particularly in the hippocampus, which is the main cause of impaired learning and memory in smokers (Durazzo et al., 2010; Sharma and Brody, 2009; Azizian et al., 2009). One possible association between smoking and AD is the APOE4 genotype (Angelopoulou et al., 2021; Rusanen et al., 2010). The highest risk was observed among the carriers of the APOE ε4 allele in the population of smokers, which is involved in the pathogenesis of AD. In addition, smoking may affect the microstructural integrity of the cerebral white matter (Gons et al., 2011), the main pathophysiological basis of cerebral small vessel disease (CSVD), which is caused by the narrowing or obstruction of small blood vessels in the brain due to inflammation and/or a buildup of misfolded proteins called plaques. However, the relationship between CSVD and AD has recently been extensively explored (Kim et al., 2020). The majority of studies have shown that CSVD has a predictive effect on the risk of AD in ageing individuals. For example, patients with mild cognitive impairment and higher volumes of cerebral white matter in the parietal lobe had more advanced AD progression than those with lower volumes of white matter (Kuller et al., 2003; Hertze et al., 2013; Tosto et al., 2015). Therefore, smoking is an indirect risk factor that links CSVD to the development of AD.

## 3 Linking pulmonary syndromes to Alzheimer’s disease

Chronic obstructive pulmonary disease (COPD) is a common lung disease which limits airflow and causes breathing difficulties. Although COPD is known to be related to the development of cognitive deficits, especially the decline of executive function, attention, memory, and logical reasoning (Tondo et al., 2018), cognitive decline without obvious neurodegenerative pathology is present in people with COPD (Schou et al., 2012; Antonelli-Incalzi et al., 2006; Incalzi et al., 1993). However, patients with severe COPD are more likely to develop preclinical neurodegenerative pathologies (Tondo et al., 2018; Schou et al., 2012; Antonelli-Incalzi et al., 2006; Incalzi et al., 1993). In addition, patients with AD and COPD have shown worse scores on executive function screening than patients with AD alone and showed a higher incidence of depressive symptoms (Tondo et al., 2018). Another study showed that young geriatric patients with COPD or asthma that caused chronic obstruction of lung function were at an increased risk of dementia in their geriatric age (Rusanen et al., 2013). The potential reason may be that in patients with asthma, some T_H_17-type inflammation-related genes, such as NOTCH1, VEGFA, and LIF, are present in the lungs and CNS. Asthma-related airway inflammation is linked to emotion-related neurological functions by increasing the expression of these genes, providing a specific inflammatory pathway that has implications for long-term brain health in depression and neuroinflammation (Dill-McFarland et al., 2024). Sleep-disordered breathing is a common airway reactivity syndrome with unclear pathogenesis. Recently, obstructive sleep apnoea syndrome (OSAS) was identified as a risk factor for the development of cognitive impairment (Buratti et al., 2017; Zhang et al., 2019). OSAS patients had noticeable Aβ changes and increased levels of P-tau in cerebrospinal fluid (Kadotani et al., 2001). Additionally, the identified risk factor APOE-ε4 gene of AD is also able to predispose to the OSAS syndrome.

## 4 Ozone, a risk environmental factor through the lung–brain axis, contributes to Alzheimer’s disease

**Figure 1:**
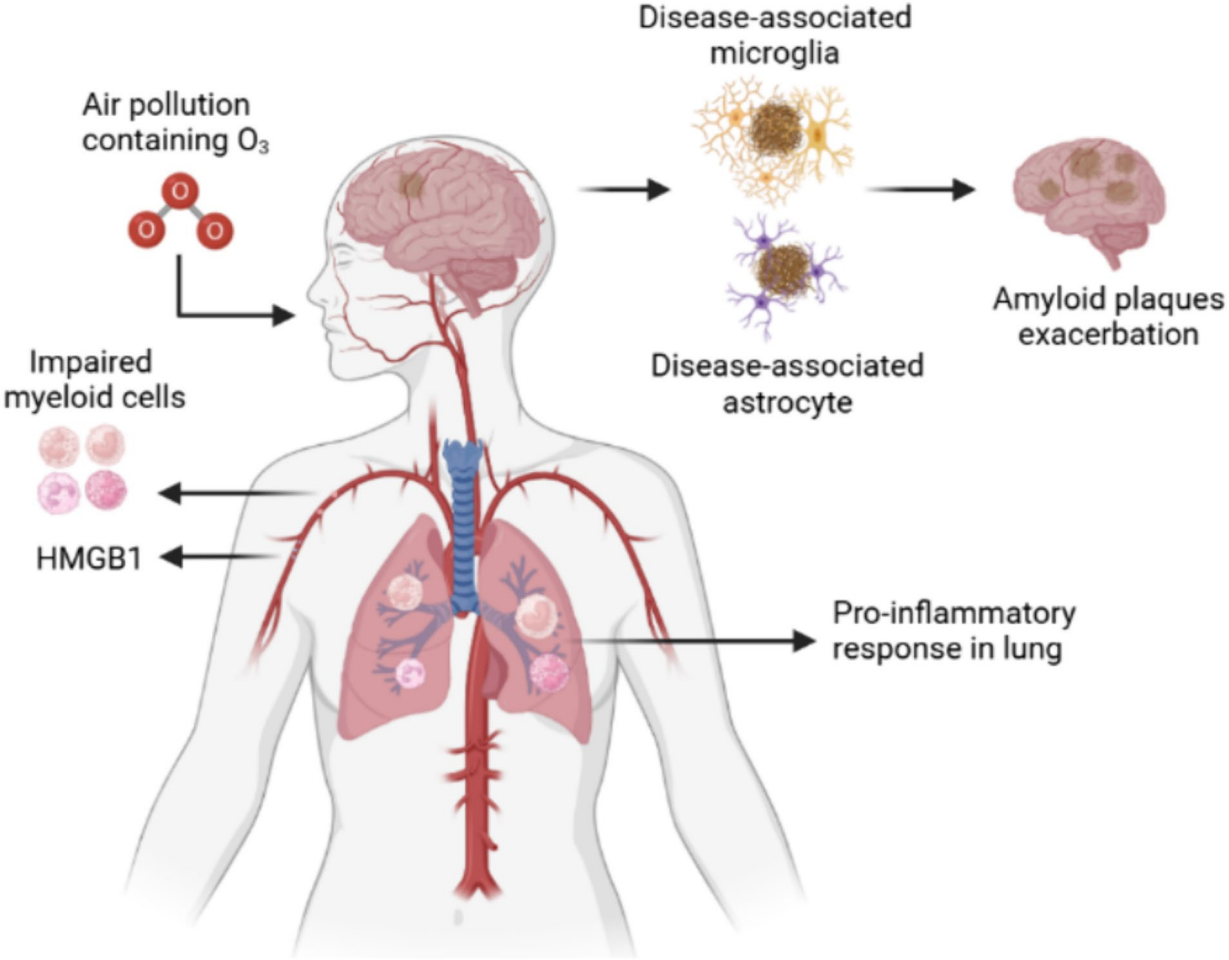
Ozone exacerbates AD by affecting the lung-brain axis by damaging microglia and astrocytes. Ozone, a reactive gas component of urban air pollution, damage lungs via the respiratory tract to produce inflammatory cells. The peripheral inflammation induced dysfunctional disease associated microglia and astrocyte to exacerbate the amyloid plaques accumulation.

Recent epidemiological studies have shown that air pollution promotes AD risk and cognitive decline in environmental factors (Mumaw et al., 2016). Air pollution in urban areas is a complex mixture of chemicals, dust, and other airborne components. For example, ozone is a widespread and chronic exposure with health effects that affect multiple organs, especially as a solid risk factor for cognitive deficits, memory decline, and AD. The ozone can increase the risk of cognitive impairment by 10.4% through various molecular mechanisms, such as enhanced oxidative stress, mitochondrial metabolism dysfunction, and neuronal cell damage (Greve et al., 2023). Ozone-induced central nervous system damage in a particular pathway named “lung–brain axis,” as ozone is a highly reactive gas and fails to cross the blood–brain barriers into the brain. Therefore, peripheral immune cells or secreted circulating factors contribute to ozone-induced brain damage. Recently, researchers (Greve et al., 2023) utilised spatial transcriptomics and proteomics to identify circulating high mobility group box 1 (HMGB1) as a protein upregulated only in an AD mouse model, and peripheral HMGB1 was shown to regulate brain Trem2 mRNA expression in the microglia. Additionally, it was found that HMGB1 has a close relationship with disease-associated astrocytes and that peripheral immune cells are important lung–brain axis interactors (Ahmed et al., 2024; Greve et al., 2023). When ozone decreased bronchoalveolar lavage immune cell HMGB1 expression in AD mice, the number of astrocytes around amyloid plaques significantly increased, implying the activation of disease-associated astrocytes (Figure 1).

## 5 Pulmonary microbiotas associated with Alzheimer’s disease and cognitive dysfunction

**Figure 2:**
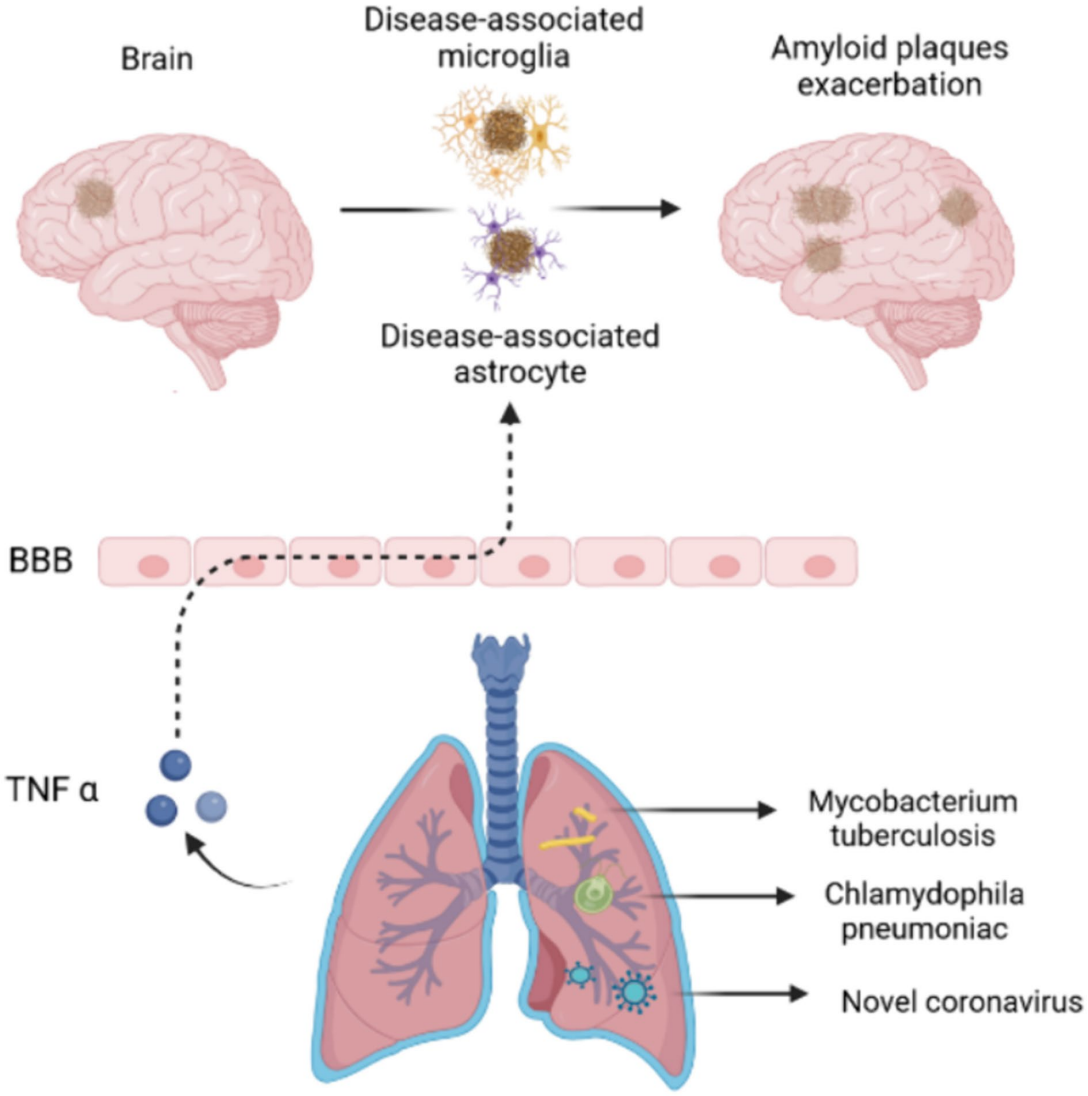
Pulmonary microbiotas associated with Alzheimer’s disease. Pulmonary microbiotas such as *Chlamydophila pneumoniae*, *Mycobacterium tuberculosis* or novel coronavirus produce the virulence factor or inflammatory reaction to damage the BBB, leading the accumulation of amyloid plaques in AD brain.

Like other organs of human body such as gastrointestinal tract, the upper respiratory tract contains a variety of bacterial species, including beneficial and pathogenic strains that was associated with individual health. The common bacteria that reside in the lungs such as Staphylococcus and Streptococcus are often detected in healthy body (Azzoni and Marsland, 2022; Li et al., 2023). A meta-analysis showed that over a five-fold increased occurrence of AD with *Chlamydophila pneumoniae* infection which is a pathogenic bacterium frequently causing pneumonia. Spirochaetal infection that also can be occurred in the lung infection had over a ten-fold increased occurrence of AD and a four-fold increased occurrence of AD in a conservative risk estimate (Maheshwari and Eslick, 2015). Additionally, some pathogenic bacteria use their own molecular mechanisms to indirectly contribute to the development of AD. For example, *Mycobacterium tuberculosis* is well-known to cause infection in the CNS which can release tumour necrosis factor α (TNF-α) (Azzoni and Marsland, 2022; Li et al., 2023). TNF-α influence the onset and development of AD in several ways: (1) TNF-α is contributing to enhance the generation of Aβ and subsequent accumulation; (2) TNF-α reduces phagocytic function of immune cells and leading the loss of neurons; (3) TNF-α rearrange the cytoskeleton to disrupt brain microvascular endothelial cells which presents the main composition of blood brain barrier through activating toll like receptor signalling. Recently, the impact of COVID-19 on cognitive function has been the focus of an increasing number of researchers. Because of long-term trajectories of cognitive change after a COVID-19 infection remain unclear, YH Liu et al. investigated cognitive changes over a period of 2.5 years among the older sufferers. The finding showed that overall incidence of cognitive impairment among older COVID-19 survivors was 19.1%. These research literatures suggests that microbial infections of the lungs are associated with AD and cognitive dysfunction (Liu et al., 2024) (Figure 2).

## 6 Exploring the potential molecular mechanisms involved in lung-brain axis in AD

Due to the importance of the lung-brain axis in physiological and pathological conditions, many studies have used the transcriptome or proteome to dissect the underlying molecular mechanisms in different disease states. Researchers commonly work with animal models, first causing damage to the lungs and then analysing the associated neurological changes in specific regions of the brain. For example, to mimic the lung inflammation in asthma model, mice were challenged with either bacterial lipopolysaccharide or ovalbumin allergens, and then the differences in the hypothalamus following lung inflammation were analyse by using RNA transcriptome profiling to investigate the neuropsychiatric sequelae (Bastawy et al., 2024). In AD-related studies, Greve et al. (2023) used spatial proteomic profiling analysis to explore dysregulated protein expression in disease-associated microglia around the amyloid beta plaques, which revealed a microenvironment-specific signature after O_3_ exposure via the lung respiration. They found that the effects of O_3_ on the brain are determined by its spatial localisation to amyloid plaques which providing us a new respective to understand the interaction of environmental factor and brain.

## 7 Discussion

The anatomical definition of the lung–brain axis refers to the autonomic nervous system, which is mainly innervated by the parasympathetic nerve (vagus nerve) and sympathetic nerve of the upper thoracic segment of the spinal cord, in which the vagus nerve plays a dominant role. However, the current broad definition of the lung-brain axis refers to the crosstalk between the lungs and brain under physiological or pathological conditions, especially pathological conditions. The lung–brain axis is involved in a wide range of neurodegenerative diseases in addition to AD, including Parkinson’s disease (Hernán et al., 2002; Ritz et al., 2014; Li et al., 2015), Multiple system atrophy et al. (Mehanna and Jankovic, 2010; Ghorayeb et al., 2004). We explored the association between the lungs and AD primarily because, among the various pathogenic mechanisms of AD, there is now an increasing interest in peripheral pathogenesis (Li et al., 2024; Zhang et al., 2024; Sirkis et al., 2024), and the lung is a peripheral organ that is closely related to the external environment.

We conducted a literature review on five aspects of lung–brain-mediated AD development: common risk factors linking the lung–brain axis, pulmonary syndromes linked to the brain, environmental factors linking the lung and brain, pulmonary microbiotas associated with AD, and the potential molecular mechanism in lung-brain axis in AD. There are several limitations to the cited studies. In recent years, the critical role of lung–brain communication has become increasingly apparent. However, the lack of research on lung-brain axis in AD leads to insufficient connections between pulmonary diseases and AD. The possible cause is that there is also a blood brain barrier between the lungs and the brain and the crosstalk of the lung to blood brain barrier remains unknow. Despite the spatial proteomic profiling analysis, there is still no single cell sequencing technology that has been applied to the lung-brain axis in AD. Another limitation in the review is that, we are more concerned about the relationship between immediate causal factors of lung disease and the pathogenesis of AD and do not account for the complexity of multifactorial lung diseases, such as lung tumours.

Overall, the involvement of the bidirectional lung–brain axis in the pathophysiology of AD may provide a new perspective on our understanding of the pathogenesis of the disease. In the future, it remains a major challenge to determine whether it is possible to treat AD by modulating the lung-brain axis. As the most common clinical medicine for AD such as Donepezil is a cholinesterase inhibitor which could exacerbation of COPD and bronchial asthma (Bonner and Peskind, 2002). Therefore, regulating the central brain function by interfering with the peripheral lungs, is a challenge and a new strategy at the same time.
